# Depressed Exercise Peak Ejection Rate Detected on Ambulatory Radionuclide Monitoring Reflects End-Stage Cardiac Inotropic Reserve and Predicts Mortality in Ischaemic Cardiomyopathy

**DOI:** 10.4021/cr203w

**Published:** 2012-07-20

**Authors:** Gian Piero Carboni

**Affiliations:** Consultant in Cardiology. Director of the Nuclear Cardiology Service, Universita Campus-Bio Medico di Roma. Via Alvaro del Portillo, 200, 00124 Roma, Italy. Email: g.carboni@unicampus.it

**Keywords:** Peak ejection rate, Ambulatory radionuclide monitoring, Vest, Ischaemic cardiomyopathy

## Abstract

**Background:**

Fifteen patients with ischaemic cardiomyopathy and inducible ischaemia were studied to determine the mechanisms of mortality. Failure of the contractile reserve during daily life activities may reflect a prognostic index.

**Methods:**

Single photon emission cardiac tomography and radionuclide ambulatory monitoring (Vest) data were analysed in all patients with a 7-year follow-up.

**Results:**

At peak exercise on Vest, the 7 non-survivors (N-SURV) showed worse peak ejection rates (PERs) and ejection fractions (EFs) compared with the 8 survivors (SURV), (2 ± 0.6 vs. 3.3 ± 0.7; end-diastolic volumes (EDVs), P < 0.003), and (34 ± 10% vs. 50 ± 13%; P < 0.02), respectively. However, exercise peak filling rates (PFRs) (1.9 ± 0.6 vs. 2.7 ± 0.9; EDVs/s) and exercise heart rates (HRs), (97 ± 17 vs. 106 ± 10), did not differ between the two groups (P > 0.05). In SURV, exercise PERs, which represented rapid left ventricular (LV) emptying, were significantly correlated with exercise PFRs, representing rapid LV filling, (r = 0.71, P < 0.04) but not in N-SURV (r = 0.66, P > 0.05). Among SURV, the Frank-Starling mechanism was thus preserved but not in N-SURV. Upon Cox analysis, overall LV function parameters, exercise PER was the only predictive measure associated with mortality (b = - 0.018, relative hazard ratio = 0.98, P = 0.02).

**Conclusions:**

Exercise PER reduced values reflected failure of the Frank-Starling mechanism, the incapacity of the heart to perform rapid contractile adaptations to daily life activities and a poor prognosis.

## Introduction

In patients with ischaemic cardiomyopathy (ICMP), survival is limited despite the improvements in revascularisation techniques, heart surgery and medical therapy. Yoon et al [1] observed that in ICMP patients who were treated with different therapeutic options, the 9-year survival was as follows: coronary artery bypass grafting, 53%; coronary artery bypass grafting plus mitral valve annuloplasty, 34%; coronary artery bypass grafting plus surgical ventricular restoration, 55%; listing for cardiac transplantation, 54%. There is, however, still a debate on the mechanism and incidence of mortality in ICMP patients with inducible ischaemia not eligible for revascularisation [2-6].

The purpose of this study was to determine in 15 patients with ICMP who were not eligible for revascularisation, the markers of mortality analysing Thallium-201 stress/rest single photon emission cardiac tomography (SPECT) and radionuclide ambulatory monitoring (Vest) data with a long-term clinical follow-up.

## Methods

### Patients

Patients with a median age of 68 years (range 62 - 73) were selected from December 2003 to July 2004 and studied with a median clinical follow-up of 7 years (range 5 - 7) during optimal medical therapy [7].

### Stress/rest SPECT

The test was performed after injection of 74 to 148 MBq (2.0 to 4.0 mCi) of Thallium-201 on a 90-degree dual-head gamma-camera (DST-XL; Sopha Medical Vision International, France), equipped with high-resolution, parallel-hole collimators. SPECT images were recorded 2 - 3 minutes after thallium-201 injection, and the baseline images were obtained 3 hours later. Thirty-two projections of 120 seconds were obtained as 64-by-64 matrices over a 180-degree orbit, with an acquisition zoom of 1.33 (pixel size, 6.8 x 6.8 mm) [8]. Bull’s eye polar maps were obtained from SPECT and the abnormal defect size (ADS) quantified as a percentage of the entire left ventricle (LV) surface. The ADS were evaluated visually [9] and as a percentage of total LV pixels and reversibility (rev) was evaluated as a percentage of ADS pixels [10]. All images were interpreted by 2 independent observers.

### Radionuclide angiography

Within 2 - 4 weeks following SPECT, the in vivo labelling of red blood cells was performed with 555 MBq (15 mCi) of Technetium-99m-sestamibi using the gamma camera (DST-XL; Sopha Medical Vision International, France) equipped with an all-purpose collimator to determine the gated equilibrium blood pool analysis (MUGA) [11] and ejection fraction (EF) for each subject. The patients were placed in the supine position, with the camera in a 30- to 45-degree left oblique projection, with a 5- to 10-degree caudal tilt. Images were gated at 16 frames/cycle by using an R-wave trigger, and 350,000 counts were obtained for each frame, with a zoom factor of 2 in a 64-by-64 matrix. LV EF was calculated by using standard, automatic, commercially available software (Sophy NXT, Software revision 2.01, Sopha Medical Vision International).

### Vest recordings

Immediately after MUGA, the Vest garment was placed over the subject's chest. The Vest detector (Capintec, Inc., 6 Arrow Road Ramsey, NJ, 07446, USA) was positioned under the gamma camera control as previously described in detail [12]. The Vest radionuclide recorder was embedded on the garment worn by patients during daily activities. The EF was calculated continuously with a Holter digital recorder using the formula of end-diastolic volumes per second (EDV/s) counts-end-systolic volumes per second (ESV/s) counts/EDV/s counts-background/s counts x 100. The peak ejection rate (PER), obtained from the systolic phase of the time-activity curve, determined the maximum rate of LV emptying, which was measured as EDV/s. The peak filling rate (PFR), calculated from the diastolic phase of the time-activity curve, revealed the maximum rate of filling in the initial rapid filling period as EDV/s. The Vest recording was ambulant in all patients. All values were recorded simultaneously, including heart rate (HR), ST trends and arrhythmias, under resting conditions (rest) and during exercise. The exercise regimen included climbing the same set of stairs located outside the hospital. As a result, the exercise oxygen uptake was uniform in all of the study subjects.

### Severity of CAD

Severity of coronary artery disease (CAD) was graded dichotomously on a scale from 0 to 1 (severe CAD = 1, in presence of occlusion of coronary bypass, Stent occlusion or triple vessel disease, moderate CAD = 0, in presence of minimal coronary obstructions, or 1-2 vessel disease).

### Follow-up

All patients were followed for a median of 7 years, (range 5 - 7.3 years) with a median of 14 medical visits (range 4 - 18) and a median of 6 hospital readmissions (range 4 - 10) for clinical instability.

### Statistical analyses

Tests were performed using a MedCalc Software (Broekstraat 52, 9030 Mariakerke, Belgium). The patients ages, Vest recording time and the follow-up duration were analysed as the median ± interquartile ranges; the SPECT, Vest and follow-up results as the means ± standard deviation and, when necessary, with unpaired or paired t-tests, Bland-Altman plots, receiver operating characteristic (ROC) curves, Cox stepwise proportional-hazards regression and Kaplan-Meier analyses. A P-value less than 0.05 was considered statistically significant. This investigation conforms with the principles outlined in the Declaration of Helsinki. A formal ethics review committee approved this protocol of study, and all subjects provided written informed consent to participate in the study.

## Results

### Stress/rest SPECT

The SPECT documented ADS and rev ADS of the extent of a mean of 28 ± 16% and 12 ± 24% pixels, respectively. The extent of ADS and the severity of the CAD were significantly correlated (r = 0.66, P < 0.008). When non-survivor patients (N-SURV) and survivor patients (SURV) were compared, the ADS extent did not differ significantly (35 ± 17 vs.21 ± 5; pixels, p > 0.05). ADS rev was documented in 71% (5/7) and 37% (3/8) of N-SURV and SURV cases, respectively (P > 0.05). Detailed indications of patients clinical characteristics, the SPECT scan results, CAD severity and NYHA class are described in Table 1.

**Table 1:** Patient Clinical Characteristics

Patient/age	MI site/LV structural damage	Number of coronary arteries with CAD/ extent of coronary obstructions	ADS extent as % of LV total pixels	ADS rev as % of ADS pixels	New York Heart Association classification	Follow-up results
1/62	Ant	3/3 by-pass/occluded only graft to LAD	45	9	2	N-SURV (Fatal MI)
2/62	Ant/prior LV aneurysm resection	2/occluded LAD/ LCX with 90% stenosis	60	2	3	N-SURV (HF)
3/69	Ant, Inf, Lat	3/patent stent to RCA and LCX, LAD with 50% stenosis	49	0	3	SURV
4/76	Ant, Inf	1/occluded stent to LAD	29	55	3	SURV
5/74	Ant, Inf, Lat /LV aneurysm	1/occluded LAD	11	0	2	N-SURV (HF)
6/74	Ant	3/3 by-pass with only occluded graft to LAD	25	10	3	N-SURV (HF)
**7/78	Ant	1/LAD with 30% stenosis	15	82	2	SURV
8/72	Inf/valvular aortic stenosis	coronary arteries with minimal obstructions	8	0	2	SURV
9/53	Ant, Inf, Lat	coronary arteries with minimal obstructions /vasospastic angina	22	0	2	SURV
10/50	Inf	ectatic LAD	7	0	2	SURV
11/62	Ant, Inf	coronary arteries with minimal obstructions	20	5	2	SURV
**12/67	Ant	3/3 by-pass with only 2 occluded grafts	28	5	3	N-SURV (HF)
*13/68	Ant, Lat	3/severe stenosis not eligible for revascularisation	47	17	3	N-SURV (HF)
*14/68	Ant, Lat, Inf	1/patent stent to RCA	27	0	2	N-SURV
15/64	Ant	1/occluded LAD	21	0	2	SURV

*implantable cardioverter-defibrillator (ICD), ** permanent pacemaker implantation for sinus node dysfunction; coronary artery disease (CAD), myocardial infarction (MI), anterior (Ant), inferior (Inf), lateral (Lat), left anterior coronary artery (LAD), left circumflex coronary artery (LCX), right coronary artery (RCA), heart failure (HF).

### Radionuclide angiography

**Figure 1:**
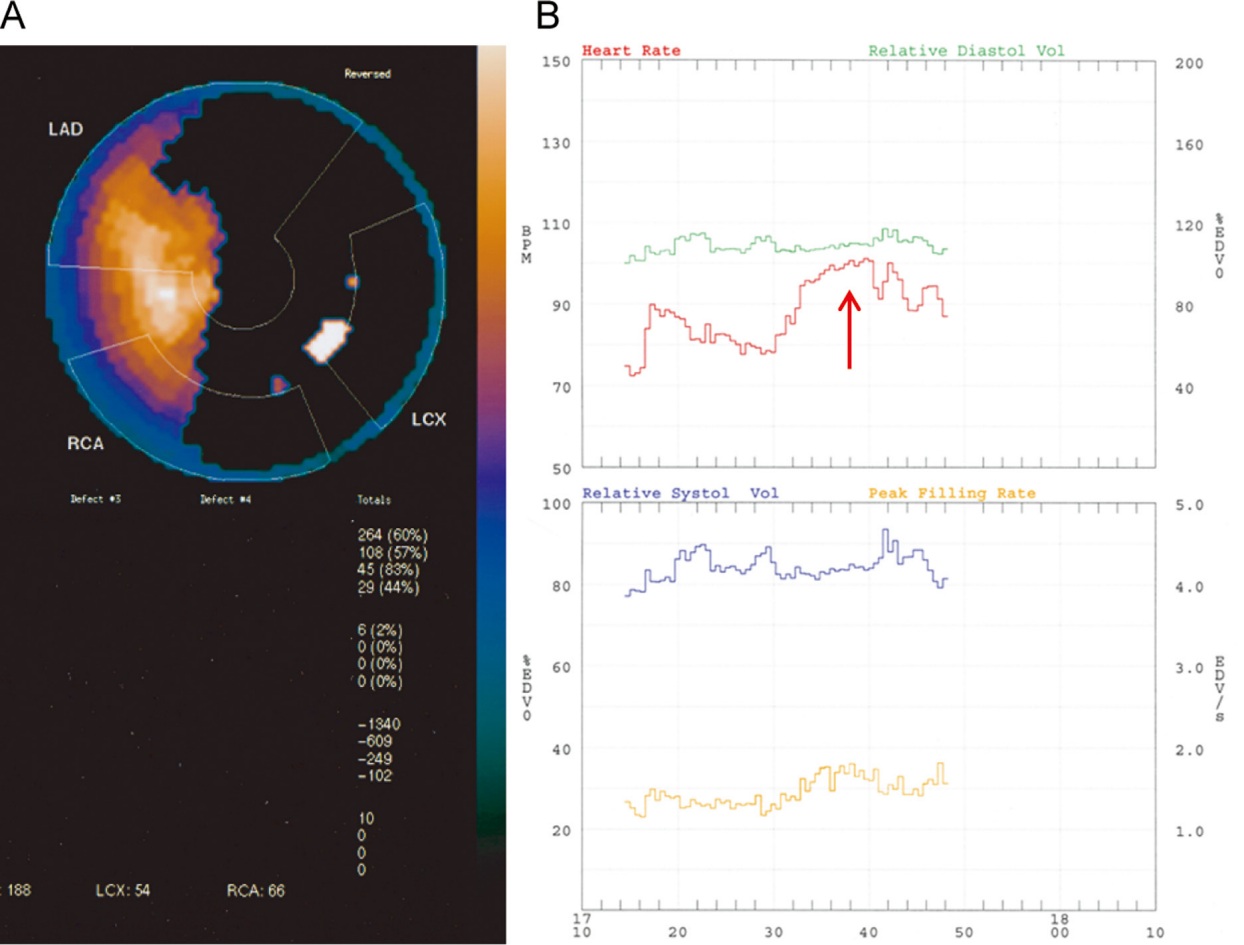
The radionuclide data of a 62-year-old man who had undergone LV aneurysmectomy and presented with occlusion of the anterior descending coronary artery, 90% stenosis of the circumflex and the right coronary artery free of obstructions. This patient manifested a 6-month mortality. A) Patient’s polar map from SPECT representing LV total pixels. The black spot represents the ADS size with an extent of 60% of the LV total pixels; the white spot represents reversibility with an extent of 2% of total ADS pixels. B) The panel shows, in this same subject, 26 min continuous ambulatory radionuclide monitoring recordings of heart rate, systolic and diastolic volumes and peak filling rate trends, at resting conditions and at peak exercise. At peak exercise (red arrow), the increment in heart rate was not associated with according changes in the systolic and diastolic volumes and peak filling rate. These data imply failure of the Frank-Starling mechanism and explain the mechanisms underlying failure of the contractile reserve and the patient’s outcome.

EFs recorded with Vest and MUGA values (31 ± 10% vs. 29 ± 10%, P > 0.05) did not differ, with a mean difference of only 3% on the Bland-Altman plot. Vest recordings lasted for a median time of 251 min (range 168 - 275). During Vest recordings, in N-SURV patients, only exercise heart rates HRs and exercise EDVs did not increase from rest, a representative example is given in Figure 1; in SURV patients, only exercise EDVs did not increase from rest; at rest, N-SURV showed lower PERs and EFs and higher ESVSs compared with SURV; at peak exercise, N-SURV showed lower PERs and EFs compared with SURV. In SURV patients, the exercise PER changes correlated with the exercise PFR changes (r = 0.71, P < 0.04), but this was not the case in N-SURV patients (r = 0.66, P > 0.05). When using the Vest, neither life-threatening arrhythmias nor significant ST depression or angina episodes were recorded. All of the Vest results are summarised in detail in Table 2.

**Table 2:** The LV Function Parameters Recorded With Vest

	N-SURV			SURV			N-SURV vs. SURV	
	rest	P	exercise	rest	P	exercise	rest P	exercise P
HR/bpm	88 ± 11	0.2	97 ± 17	83 ± 12	0.001	106 ± 10	0.4	0.2
EDV(mL)	105 ± 5	0.2	97 ± 16	102 ± 5	0.7	105 ± 22	0.2	0.4
ESV(mL)	80 ± 8	0.02	65 ± 20	65 ± 11	0.02	52 ± 16	0.008	0.2
% EF	25 ± 7	0.01	34 ± 10	38 ± 9	0.01	50 ± 13	0.01	0.02
PFR(edv/s)	1.3 ± 0.3	0.01	1.9 ± 0.6	1.3 ± 0.4	0.01	2.7 ± 0.9	0.9	0.06
PER(edv/s)	1.4 ± 3	0.01	2 ± 0.6	2.3 ± 0.6	0.01	3.3 ± 0.7	0.003	0.003

### CAD severity

Severe CAD characterised 86% (6/7) and 25% (2/8) of the N- SURV and SURV patients, respectively (P < 0.04).

### Follow up

Heart failure was observed in 47% (7/15) of patients during follow up. Overall, 1 patient died of fatal myocardial re-infarction after 6 months, and 6 patients died of heart failure after a median of 5.8 years (range 5 - 7 years). Among the N-SURV patients, only two underwent the implantation of a cardioverter defibrillator (ICD), and only 1 was treated with a permanent pacemaker for sinus node dysfunction (SND). Among the SURV patients, only1patient was treated with the implantation of a permanent pacemaker for SND. There was no indication for the treatment of any patient with a biventricular pacemaker.

**Figure 2:**
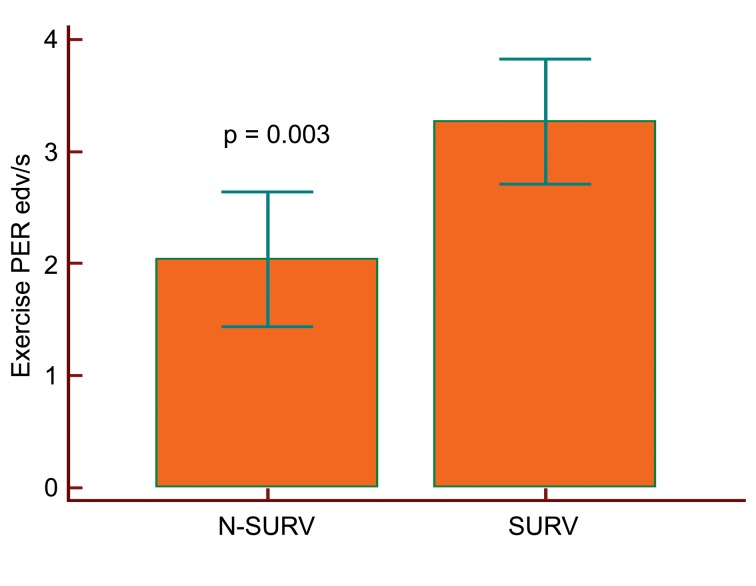
Compared to SURV patients, N-SURV patients showed lower exercise PERs with minimally overlapping values.

**Figure 3:**
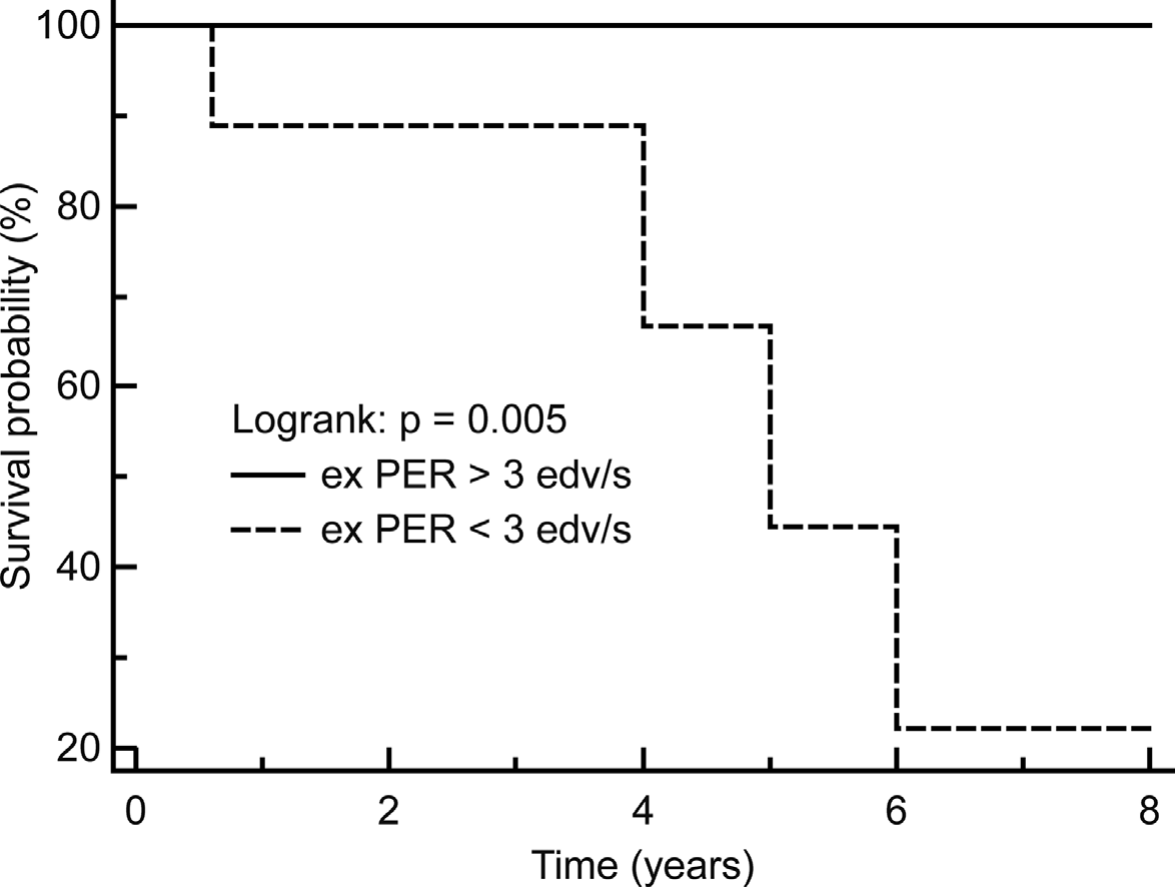
Kaplan-Meier curves; exercise (ex) PER values stratified individuals with respect to mortality.

In the Cox proportional hazards regression model, which allows for the analysis of the effects of several risk factors on survival, the exercise PERs were the only significant predictive measures of mortality (b = - 0.018, P = 0.02, relative hazard ratio = 0.98, % confidence interval (CI) 0.97 to 0.99). Resting and exercise EFs and PFRs, resting PERs on Vest recordings, the angiographic extent of CAD and the extent of ADS and ADS rev (ischaemia) on thallium-201 SPECT were not predictive of mortality. The results did not change after adjustment for the functional capacity measured by NYHA class, age and established cardiovascular risk factors (P > 0.05 for all). Compared to SURV patients, N-SURV patients showed significantly lower PERs with a minimal overlapping of the values (2 ± 0.5 vs. 2.7 ± 0.4 EDV/s, P = 0.003, 95% CI of the difference 0.4 to 1.9, Fig. 2). Analysing the relationship between exercise PERs and death also with ROC curves, exercise PERs showed under the ROC curve an area of 0.89, P = 0.0001, with a criterion value < than 3 EDV/s. This cutoff value stratified individuals in the Kaplan-Meier survival curve analysis with respect to mortality (Fig. 3).

## Discussion

The finding of low exercise PER values resulted the strongest predictor of mortality. Because the EF [13, 14] is the most used prognostic parameter in ICMP in clinical practice, these results require further consideration. In healthy hearts, an enhanced relaxation allows rapid filling and increased contractility. This observation constitutes the basis of the Frank-Starling law, which asserts that the force of the LV contraction is a function of the end-diastolic length of the cardiac muscle, which is strongly related to the EDV increase [15]. Conversely, in both N-SURV and SURV patients, EDVs did not increase accordingly to the workload increment on exercise. Only in N-SURV, the exercise PERs and exercise PFRs, representing rapid LV emptying and relaxation and filling, did not interact. In N-SURV, IN addition, the exercise PERs and EFs were significantly lower with respect to SURV. This phenomenon indicated thus the end-stage failure of the Frank-Starling mechanism with exhausted inotropic reserve in N-SURV patients [16]. The evaluation of PER during daily life activity may therefore be an adjunct parameter to EF values for the indication of multiple classes of drugs or drugs with combined effects to increase the output and decrease the filling pressure [17] in patients predisposed to heart failure, which causes significant morbidity and carries a 50% 5-year mortality [18].Moreover, this study began in 2003 and 2004, when the indications for ICD treatment differed from the currently used criteria [19]. Practice surveys have shown a persistent under-utilisation of ICD treatment. For every ICD implant in European countries, there are four implants in the United States. This disparity is attributable to the relative paucity of diagnostic resources rather than the use of different guidelines [20]. Vest recording of LV function parameters during daily activities could be a useful technique for better tailoring medical therapy and for improving decision making for the potentially lifesaving ICD implantation in certain patients not eligible for revascularisation.

### Conclusions

The exercise PER reduced values are thus an important expression of failure of myocardial contractile reserve and depict the incapacity of the heart to perform rapid contractile adaptations to daily life activities and are related to poor prognosis.

The prognostic value of the PER low response to exercise during daily activities appears thus superior to the exercise EF in patients with ICMP not eligible for revascularisation.

Radionuclide ambulatory monitoring may serve as a valuable adjunct in the non-invasive evaluation of LV function of high selected groups of patients with ICMP who need further cardioprotective actions [21-23] besides optimal medical therapy.

Our study has however some limitations due to the small patient sample. The patient population in our study was also quite heterogeneous for CAD severity, which may explain the depressed PER values. In addition, PER and PFR may vary widely from patient to patient due to patient selection or technical reasons [24]. Despite these limitations, the PER and PFR abnormal values assessed by other authors [25] and those observed in this study are significantly correlated. Our findings thus have a reasonable and consistent clinical validity. However, while very interesting and suggestive these results will need to be confirmed in a much larger trial.
